# Case report: Transplantation of human induced pluripotent stem cell-derived cardiomyocyte patches for ischemic cardiomyopathy

**DOI:** 10.3389/fcvm.2022.950829

**Published:** 2022-08-16

**Authors:** Shigeru Miyagawa, Satoshi Kainuma, Takuji Kawamura, Kota Suzuki, Yoshito Ito, Hiroko Iseoka, Emiko Ito, Maki Takeda, Masao Sasai, Noriko Mochizuki-Oda, Tomomi Shimamoto, Yukako Nitta, Hiromi Dohi, Tadashi Watabe, Yasushi Sakata, Koichi Toda, Yoshiki Sawa

**Affiliations:** ^1^Department of Cardiovascular Surgery, Osaka University Graduate School of Medicine, Suita, Japan; ^2^Center for iPS Cell Research and Application, Kyoto University, Kyoto, Japan; ^3^Department of Nuclear Medicine and Tracer Kinetics, Osaka University Graduate School of Medicine, Suita, Japan; ^4^Department of Cardiology, Osaka University Graduate School of Medicine, Suita, Japan

**Keywords:** human induced pluripotent stem cell-derived cardiomyocyte, ischemic cardiomyopathy, transplantation, regenerative therapy, clinical trial

## Abstract

Despite major therapeutic advances, heart failure, as a non-communicable disease, remains a life-threatening disorder, with 26 million patients worldwide, causing more deaths than cancer. Therefore, novel strategies for the treatment of heart failure continue to be an important clinical need. Based on preclinical studies, allogenic human induced pluripotent stem cell-derived cardiomyocyte (hiPSC-CM) patches have been proposed as a potential therapeutic candidate for heart failure. We report the implantation of allogeneic hiPSC-CM patches in a patient with ischemic cardiomyopathy (ClinicalTrials.gov, #jRCT2053190081). The patches were produced under clinical-grade conditions and displayed cardiogenic phenotypes and safety *in vivo* (severe immunodeficient mice) without any genetic mutations in cancer-related genes. The patches were then implanted via thoracotomy into the left ventricle epicardium of the patient under immunosuppressive agents. Positron emission tomography and computed tomography confirmed the potential efficacy and did not detect tumorigenesis in either the heart or other organs. The clinical symptoms improved 6 months after surgery, without any major adverse events, suggesting that the patches were well-tolerated. Furthermore, changes in the wall motion in the transplanted site were recovered, suggesting a favorable prognosis and the potential tolerance to exercise. This study is the first report of a successful transplant of hiPSC-CMs for severe ischemic cardiomyopathy.

## Introduction

Severe heart failure is a lethal disease with high mortality, and patients' quality of life is significantly reduced despite the development of medical treatments (1). When severe heart failure is reversible, drug treatment is prioritized; however, at the irreversible stage, a left ventricular assist device (LVAD) or heart transplantation is considered to be the first-line therapy. Heart transplantation is an extremely effective treatment for heart failure that can prolong life expectancy. However, a shortage of donors worldwide is a major drawback, and securing donors is expected to be increasingly challenging in the future (2). For LVAD, destination therapy or bridge-to-transplant is performed; however, complications such as infections and cerebral thrombosis are major problems (3). Therefore, there is a need for the development of new treatments that can replace artificial hearts and heart transplants for irreversible stages of heart failure and therapies that prevent the progression of heart failure toward the irreversible stage. Considering these challenges, the expectations for regenerative medicine are increasing.

In recent years, although cytokine-based angiogenesis treatment using somatic stem cells for heart failure has been performed, it is considered that angiogenesis-induced activation in the hibernating myocardium does not provide sufficient clinical effects in cases of severe cardiomyopathy wherein a large number of functional cardiomyocytes have been lost. In such cases, it is necessary to generate cardiomyocytes externally, transplant them into the failing heart, and electrically and functionally integrate them with the recipient heart.

The generation of a large number of new cardiomyocytes, followed by their integration within recipient hearts, is a promising treatment for severely damaged myocardia with few cardiomyocytes. Recently, induced pluripotent stem cells (iPSCs) have been developed from human somatic cells. iPSCs have the property of being able to differentiate into cells of all body organs and are expected to be a source of cell therapy for various diseases (4, 5). Basic research studies have demonstrated that iPSCs can differentiate into cardiomyocytes as single cells or myocardial tissues (6–10). These iPSC-derived cardiomyocytes beat spontaneously and produce and release cytokines that induce angiogenesis. Thus, it is conceivable that iPSCs may be used to achieve cardiomyogenesis to form cardiomyocytes that can mechanically contract and repair myocardial tissue via paracrine angiogenic factors.

Here, we report the case of a 51-year-old male patient with ischemic cardiomyopathy who had already received maximum anti-heart failure medications such as digitalis, diuretics, angiotensin-converting-enzyme (ACE) inhibitors, angiotensin receptor blockers, beta-blockers, anti-aldosterone drugs, and oral cardiotonic. He was successfully treated with clinical-grade human induced pluripotent stem cell-derived cardiomyocyte (hiPSC-CM) patches with typical cardiogenic phenotypic properties in a first-in-human clinical trial.

## Case description

**Figure 1:**
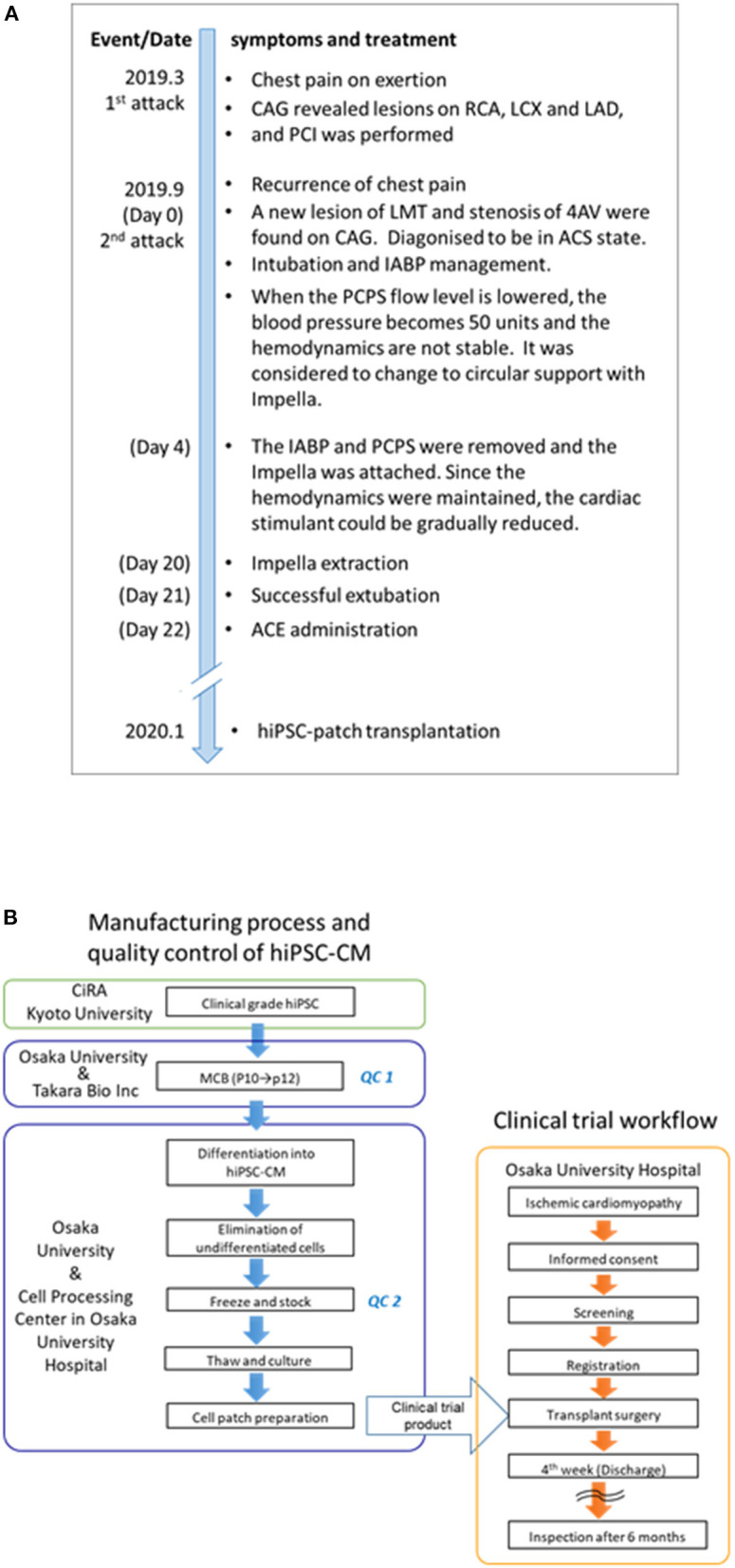
Timeline of symptoms and treatment and the clinical trial flowchart of the human induced pluripotent stem (iPS) cell-derived cardiomyocyte (hiPSC-CM) patches. **(A)** The date in parentheses indicates the number of days since the first attack. The patient underwent anti-heart failure medications (maximum dosages), such as beta-blockers, ACE inhibitors, and diuretics, before transplantation. In the first 3 months after transplantation, the patient received immunosuppressant drugs, including steroids, tacrolimus hydrate, and mycophenolic acid (mofetil); thereafter, these drugs were discontinued. Notably, no medications were introduced/stopped post transplantation. **(B)** The clinical trial flowchart of hiPSC-CM patches. Clinical-grade iPS cells were established and procured from CiRA (Kyoto University). The hiPSC-CM patches were manufactured in the cell processing center in Osaka University Hospital using the MCB cells generated in the Center for Gene and Cell Processing of Takara Bio Inc. (Kusatsu, Japan). The following quality inspections were performed during the manufacturing process: QC1: Quality check of the MCB (7). The detailed procedure of the generation of MCB is described elsewhere (7). QC2: viability, purity of cardiomyocytes, sterility, mycoplasma testing, and endotoxin testing (Supplementary Table 1). CAG, coronary angiography; RCA, right coronary artery; LCX, left circumflex coronary artery branch; LAD, left anterior descending coronary artery; PCI, percutaneous coronary intervention; LMT, left main coronary trunk; AV, atrioventricular node branch; ACS, acute coronary syndrome; IABP, intra-aortic balloon pumping; PCPS, percutaneous cardiopulmonary support; and ACE, angiotensin-converting enzyme; iPS, induced pluripotent stem; hiPSC-CM, human induced pluripotent stem cell-derived cardiomyocyte; MCB, master cell bank.

A 51-year-old male patient was repeatedly admitted to the hospital owing to severe heart failure due to ischemic cardiomyopathy (Figure 1A). In 2019, he suffered an acute myocardial infarction and was treated via percutaneous coronary intervention (PCI) targeting the three major coronary arteries. Six months after treatment, he complained of chest pain. A second acute myocardial infarction episode was diagnosed; PCI was introduced via intra-aortic balloon pumping (IABP), and percutaneous cardiopulmonary support was administered due to ventricular fibrillation. However, his condition worsened, and catecholamine and Impella® were then used as therapeutic agents. Although catecholamine treatment and circulatory support could eventually be withdrawn on day 21 after the second attack (Figure 1A), the heart failure persisted (New York Heart Association [NYHA] classification = III). Ultrasonography revealed severe asynergy throughout the wall of the left ventricle (LV) (ejection fraction [EF] = 30%). Positron emission tomography (PET) further revealed ischemia in the middle-apex (anterior wall) and the base-apex (posterior-lateral wall); an infarcted area in the inferior wall was observed despite the absence of significant stenosis in all coronary arteries. The patient was then treated with anti-heart failure medications (maximum dosages), such as beta-blockers, ACE inhibitors, and diuretics. Finally, hiPSC-CM transplantation was considered a therapeutic option.

The regenerative treatment protocol (Japan Registry of Clinical Trials, https://jrct.niph.go.jp/en-latest-detail/jRCT2053190081, Figure 1B) using allogeneic iPSC-CM patches was approved by the institutional review boards of Osaka University (#199006-A) and the Ministry of Health, Labor, and Welfare, Japan (#2019-143). The transplantation procedure was performed after obtaining written informed consent from the patient and his family. The study was conducted in accordance with the principles of the Declaration of Helsinki.

## Properties of hiPSC-CM patches used for transplantation

The methods for hiPSC culture, cardiomyogenic differentiation, purification, and cell patch preparation as well as details regarding the *in vivo* tumorigenicity assay are described in the Supplementary Methods. The detailed characterization of the hiPSCs and the master cell bank (MCB) are presented elsewhere (7) and in the Supplementary Data (Supplementary Figure 1 and Supplementary Table 1), respectively. The hiPSC-CM patch used in this clinical trial was prepared by a method that cleared the tumorigenicity denial test earlier (7). The hiPSC-CM cells used passed all quality inspections, as shown in Supplementary Table 1.

## Measurement of cardiac function

Regional myocardial displacement was assessed using four-dimensional computed tomography (4DCT), as described in the Supplementary Methods. The methods for evaluating end-systolic wall stress (ESS), tumor formation, and myocardial blood flow (MBF), including the acquisition of PET/computed tomography (CT) imaging, are also described in Supplementary Methods.

## Clinical trial

**Figure 2:**
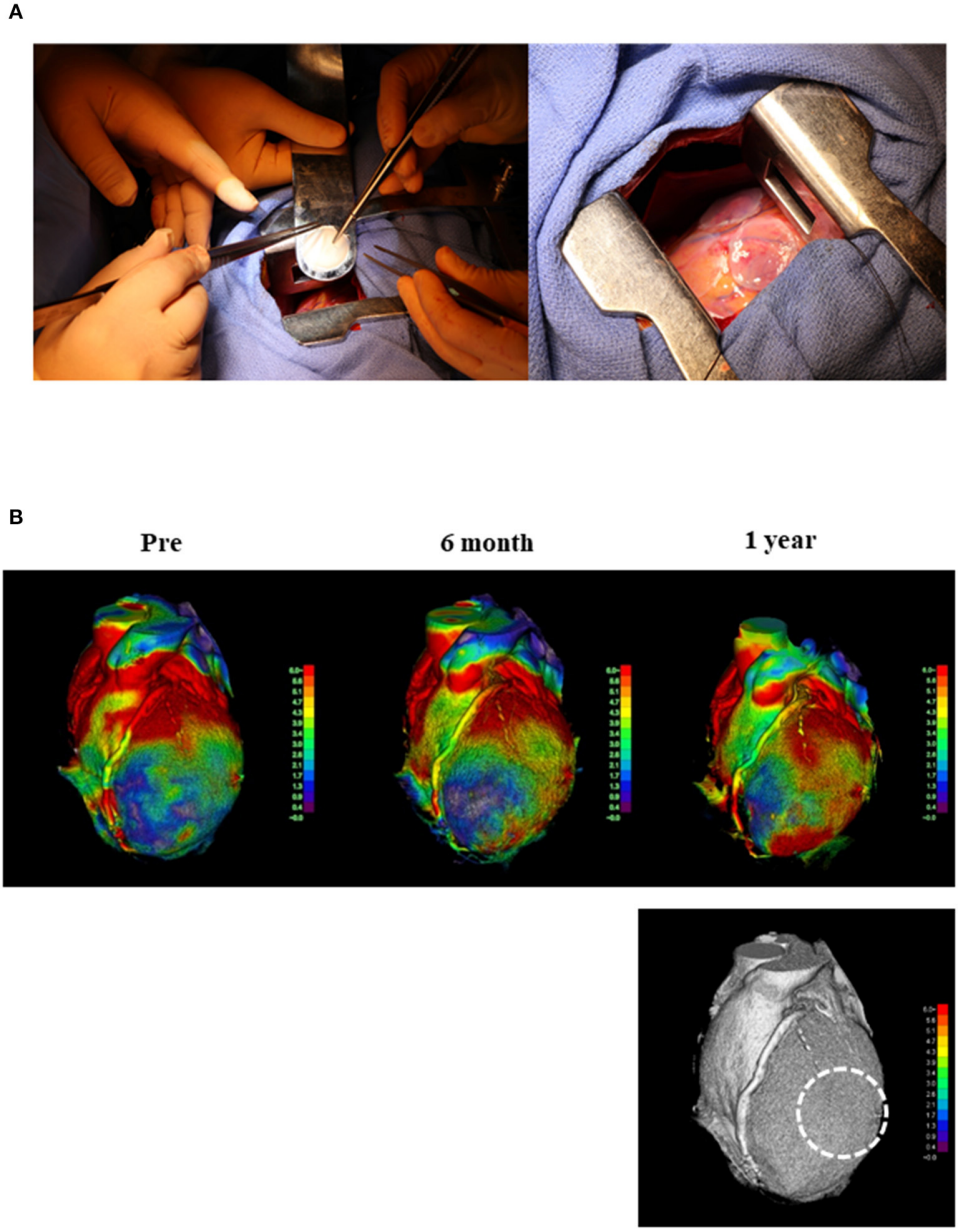
Transplantation of hiPSC-CM and cardiac moving pattern before and after surgery. **(A)** First transplantation of hiPSC-CM patches onto the heart surface of a patient with severe cardiomyopathy. Left: hiPSC-CM patch was transplanted into the epicardium of the anterior and lateral walls of the LV through the fourth intercostal space under IABP support. Right: Three patches were transplanted into the epicardium of the LV anterior and lateral walls. **(B)** The moving pattern observed via four-dimensional CT. Colors were set so that red indicated a good dynamic area; the darker the color, the lower the movement. The images show the moving pattern in chronological order of the heart at pre-implantation (pre), 6 months, and 1 year after implantation. The dotted line in the bottom figure shows the area where the patches were attached. hiPSC-CM, human induced pluripotent stem cell-derived cardiomyocyte; LV, left ventricle; IABP, intra-aortic balloon pumping; CT, computed tomography.

We prepared three allogeneic iPSC-CM patches (3.3 × 10^7^ cells/patch) that were successfully transplanted into the epicardium of the LV anterior and lateral walls (Figure 2A) through the fourth intercostal space under IABP support; no other surgical procedures, including coronary artery bypass grafting, were performed. The IABP support was withdrawn the day after surgery, and the catecholamine infusion was discontinued 8 days post transplantation. During the first 3 months after transplantation, the patient received immunosuppressant drugs, including prednisolone, tacrolimus, and mycophenolate mofetil; thereafter, these drugs were discontinued (Supplementary Figure 2). The patient underwent standard rehabilitation after surgery. Arrhythmia was constantly monitored with a 24-h electrocardiogram (ECG) monitor, and Holter ECG was also measured at the required timing to check for arrhythmias. The cardiac function and myocardial blood perfusion were measured using cardiac CT, wall stress measurements, and MBF PET. Tumorigenesis (safety) was investigated via fluorodeoxyglucose-PET (FDG-PET).

The patient was extubated the day after surgery, stayed in the ICU for 3 days, including the day of surgery, and was discharged 1 month after surgery. He underwent standard rehabilitation after surgery.

We did not detect any complications, including arrhythmias, tumor formation, or immunosuppression-related severe adverse events. Importantly, the patients' symptoms improved from NYHA III to II at 6 months and 1 year after transplantation (Supplementary Table 2). Enhanced CT demonstrated that the LV end-systolic volume index (LVESVI) increased after transplantation (Supplementary Table 2) but was substantially decreased 1 year after transplantation. Peak VO2 improved at 6 months and 1 year after transplantation without cardiac rehabilitation (Supplementary Table 2).

**Figure 3:**
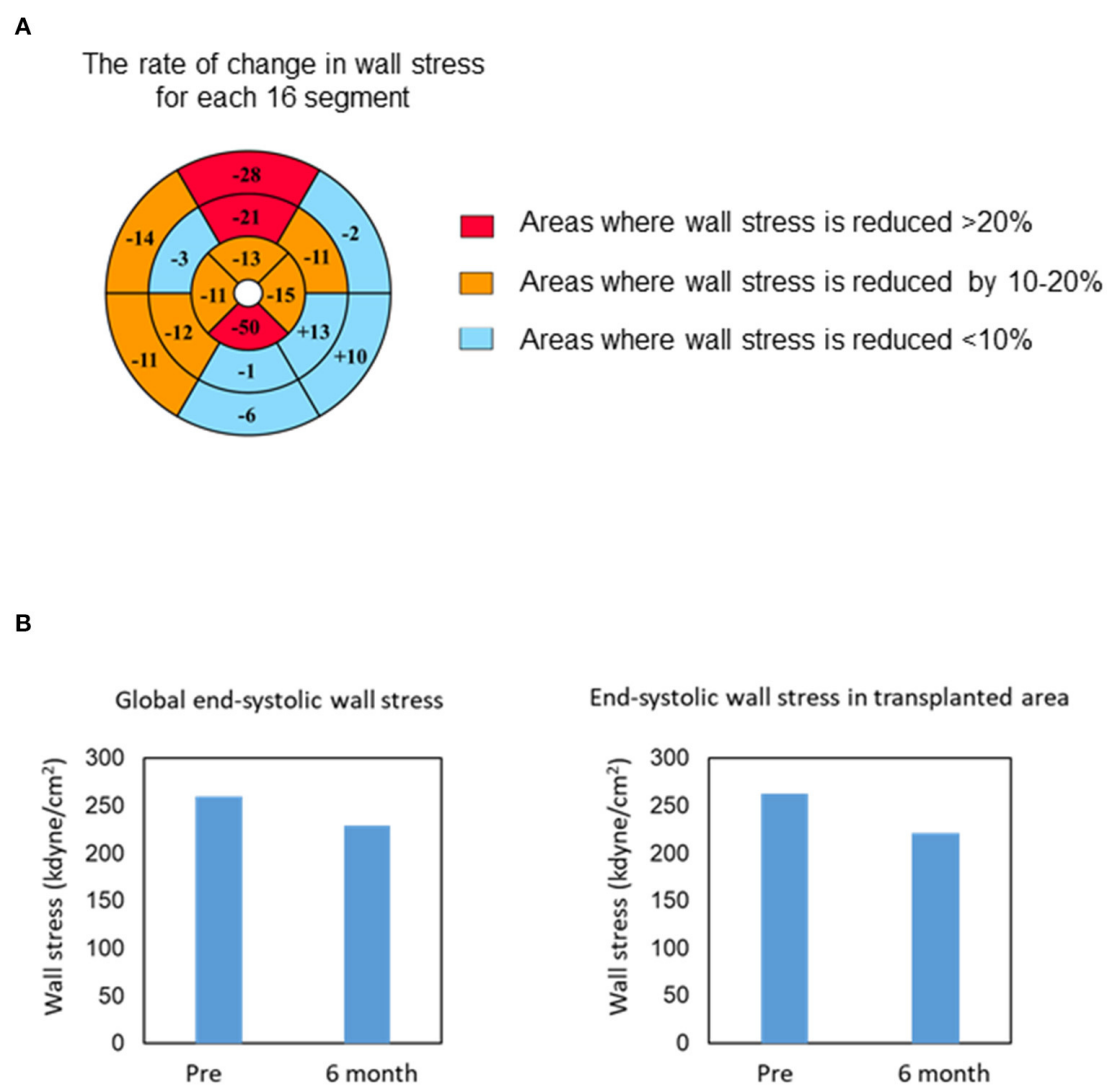
Changes in end-systolic wall stress. **(A)** Bull's-eye grid showing the changes in end-systolic wall stress before and 6 months after surgery, calculated by computed tomography. Red and orange highlight the areas of reduction in wall stress by over 20 and 10–20%, respectively. Light blue highlights the remaining areas. **(B)** Change in the global (left) and regional (transplanted area, right) end-systolic wall stress 6 months after transplantation.

Myocardial displacement was determined based on the regional myocardial wall motion in each region using a dedicated workstation comprising 320 cardiac CT images. This analysis revealed that the moving distance, especially in the transplantation area, improved significantly at 6 months and 1 year after transplantation (Figure 2B and Supplemental Movies), indicating that the myocardium under the transplanted sheets greatly benefited from the angiogenesis mediated by cytokine-paracrine effects. The LV global wall stress, especially that in the LV anterior and lateral walls that received the allogeneic iPSC-CM patches, was reduced after surgery (Figure 3), suggesting the possible attenuation of cardiac fibrosis and the prolongation of survival.

**Figure 4:**
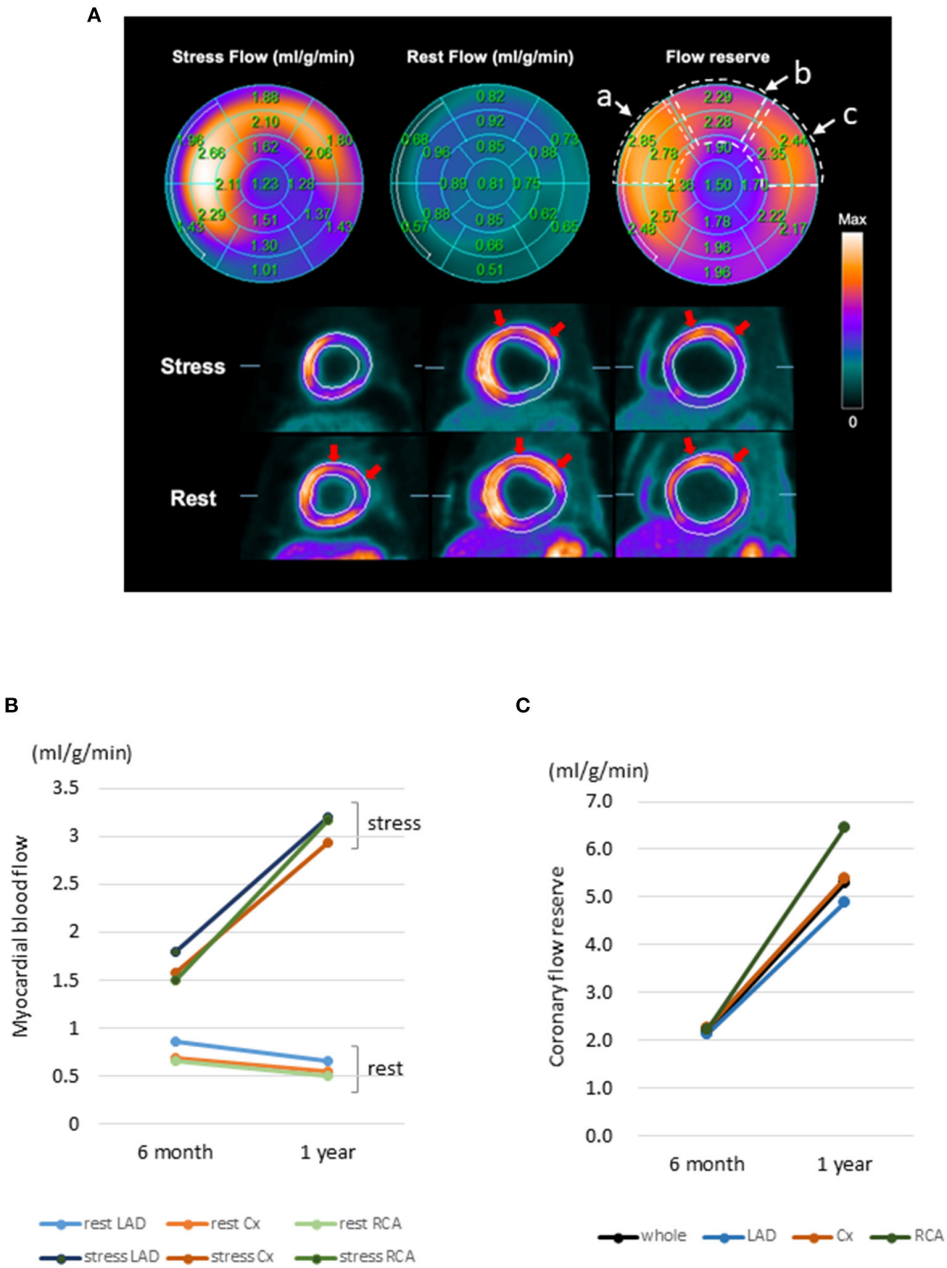
^13^N-ammonia PET images of myocardial blood flow at rest and during stress after hiPSC-CM sheet implantation. **(A)** Color-coded polar maps with superimposed 17-segment bull's-eye grids with blood flow quantitated under stress and at rest, as well as flow reserve values, are shown in each segment (upper figures). Myocardial blood flow images (short axis) are shown in the lower figures. Red arrows indicate preserved myocardial flow reserves in the anteroseptal to lateral segments. The areas surrounded by the dotted line in the flow reserve figure show a: anteroseptal, b: anterior, and c: anterolateral regions, and the average values of the two area parts were calculated. **(B)** Change in myocardial blood flow. Myocardial blood flow in the segmental regions LAD, Cx, and RCA at rest and under stress. **(C)** Change in myocardial flow reserve. Myocardial flow reserve in the whole and each segmental region at 6 months and 1 year after hiPSC-CM patch transplantation. ^13^N-ammonia PET, ^13^N-ammonia positron emission tomography; hiPSC-CM, human induced pluripotent stem cell-derived cardiomyocyte; LAD, left anterior descending coronary artery; Cx, circumflex coronary artery; RCA, Right coronary artery.

According to the ^13^N-ammonia PET images (Figure 4A), the MBF at rest was left anterior descending (LAD) 0.85, circumflex coronary artery (Cx) 0.69, and right coronary artery (RCA) 0.66 ml/g/min at 6 months after transplantation (Figure 4B). However, 1 year after transplantation, the MBF at rest showed a slight decline (LAD 0.65; Cx 0.54; RCA 0.50 ml/g/min) (Figure 4B). The MBF under stress was LAD 1.80, Cx 1.58, and RCA 1.49 ml/g/min at 6 months after transplantation but improved 1 year after transplantation (LAD 3.19; Cx 2.93; RCA 3.17 ml/g/min) (Figure 4B). Moreover, from 6 months to 1 year after transplantation, the coronary flow reserves in the whole myocardium greatly improved from 2.16 to 5.30, and the coronary flow reserves in all segmental regions of LV were also dramatically ameliorated (6 months vs. 1 year: LAD 2.12 vs. 4.90; Cx 2.27 vs. 5.40; RCA 2.21 vs. 6.47) (Figure 4C). Furthermore, changes in LV wall motion in the transplanted site were quite dramatic comparing the values at rest with those under stress conditions (Supplemental Movies), and the coronary flow reserve values were similar to those in healthy individuals. Overall, this result suggests a favorable prognosis and the potential tolerance to exercise. Notably, no medications were introduced or stopped post transplantation. Consistent with the potential tumorigenic safety reported in the preclinical study, the FDG-PET analysis revealed no tumorigenesis after transplantation (Supplementary Figure 3).

## Discussion

Here, we report the first-in-human trial of clinical-grade hiPSC-CM patches to treat ischemic cardiomyopathy in a patient and describe its potential efficacy and safety. Shiba et al. (11) reported that arrhythmias markedly increased in the early phase after transplantation of hiPSC-CMs using a needle. By contrast, we did not detect lethal arrhythmias or tumorigenesis after transplantation in the clinical case. Moreover, our preclinical data (7) also revealed that the clinical-grade hiPSC-CM patches are non-tumorigenic and non-arrhythmogenic and might be a safe methodology to deliver cardiac cells. Therefore, we assume that the reported arrhythmias did not occur due to transplantation of cardiomyocytes, but rather due to the cell introduction method, particularly due to the needle injection; nonetheless, further clinical evaluations are needed for safety assurance (11, 12).

The major question in the context of hiPSC-CM patches is whether the transplanted cardiac tissues may be electrically integrated within the recipient's heart, i.e., whether they can undergo “cardiomyogenesis” in a damaged heart with few functional cardiomyocytes. Transplanted cardiomyocyte patches can reportedly undergo contraction/relaxation (13) or repeated electrical potential activation (14) in the recipient heart in a synchronous fashion. The cardiogenic properties (both histological and functional) of cardiomyocyte patches were also demonstrated in our preclinical study (7) (Supplementary Figure 1). Importantly, in the preclinical study, we observed that the transplanted cardiac tissues facilitated functional recovery; however, the extent of the mechanical contribution of the transplanted cardiomyocytes to the contractile force of the diseased heart needs further investigation.

The functional recovery may also depend on angiogenesis, which positively impacts the hibernating myocardium (15, 16). In particular, the generation of new functional blood vessels, as we detected in the preclinical experiments (7), may be sufficient to allow effective perfusion of blood to hibernating myocytes and improve the coronary flow reserve in damaged hearts. Based on the *in vitro* cytokine expression profile, the angiopoietin family may have a role in the maturation of blood vessels (7). The decrease in the resistance of the peripheral coronary arteries (7) may contribute to the improvement of the global coronary flow reserve in the ischemic myocardial tissue and may lead to tolerance to exercise.

In this study, PET detected improved LV wall motion upon transplantation of the patches, especially under stress, accompanied with ameliorated coronary flow reserve, suggesting a potential improvement in exercise tolerance. Furthermore, our PET study revealed that MBF at rest gradually decreased, suggesting that the myocardium could function under low blood flow at rest. The damaged myocardium showed time-dependent recovery after transplantation.

In this clinical trial, we suspended the administration of immunosuppressant drugs 3 months after transplantation. Notably, we could not clearly elucidate whether transplanted patches survived in the human heart. However, we speculate that the changes in cardiac function, as well as the preserved coronary flow reserve, were mainly dependent on the angiogenic action, which was sufficient to achieve the clinical needs in the treatment of severe heart failure. Further research is warranted to increase the effectiveness of hiPSC transplantation therapy. For instance, a method of monitoring cell survival after transplantation, a detailed analysis of the immune response and immune control mechanism to establish a more appropriate immunosuppressive agent administration routine, and attempts to co-transplant hiPSCs with other types of cells (17) or tissues (18) to enhance cardiomyogenesis should be considered.

## Data availability statement

The original contributions presented in the study are included in the article/Supplementary material. Further inquiries can be directed to the corresponding author.

## Ethics statement

The studies involving human participants were reviewed and approved by the Institutional Review Boards of Osaka University (#199006-A) and the Ministry of Health, Labor, and Welfare, Japan (#2019-143). The patients/participants provided their written informed consent to participate in this study.

## Author contributions

SM: project planning, experimental design, clinical studies, data analysis, and manuscript writing. SK, TK, YSak, and KT: conducting clinical studies. KS, YI, and TW: clinical data collection and analysis. HI, EI, and MT: preparation and quality control of clinical trial products. MS: coordinating the clinical trial. NM-O: editing of manuscript drafts. TS and YN: coordinating clinical trials. HD: providing clinical-grade cells. YSaw: design and supervision of the entire project. All authors contributed to the article and approved the submitted version.

## Funding

This study was funded by Japan Agency for Medical Research and Development (AMED) under the Grant Numbers JP20bm0204003, JP17bk0104044, JP19bk0104002, and JP20bk0104110.

## Conflict of interest

The authors declare that the research was conducted in the absence of any commercial or financial relationships that could be construed as a potential conflict of interest.

## Publisher's note

All claims expressed in this article are solely those of the authors and do not necessarily represent those of their affiliated organizations, or those of the publisher, the editors and the reviewers. Any product that may be evaluated in this article, or claim that may be made by its manufacturer, is not guaranteed or endorsed by the publisher.

## Supplementary material

Supplementary figure 1 Characteristic properties of hiPSC-CM patch.

Supplementary figure 2 Dose of immunosuppressants, the trough value of tacrolimus, and biomarker levels of renal and liver function.

Supplementary figure 3 FDG-PET imaging at 6 months after transplantation of hiPSC-CM patch.

Supplementary table 1 Quality test of hiPSC-CMs.

Supplementary table 2 Changes in cardiac function, exercise tolerance, and heart failure classification after transplant surgery.

Supplementary video 1 4DCT movie at 6 months after transplantation.

Supplementary video 2 4DCT movie at 1 year after transplantation.

## Abbreviations

4DCT: four-dimensional computed tomography
CGH: comparative genomic hybridization
CiRA, Center for iPS Cell Research and Application: Kyoto University
CNV: copy number variation
Cx: circumflex coronary artery
cTNT: cardiac Troponin T
ESS: end-systolic wall stress
FDG-PET: fluorodeoxyglucose-positron emission tomography
H&E: hematoxylin and eosin
hiPSC-CM: human induced pluripotent stem cell-derived cardiomyocyte
IABP: intra-aortic balloon pumping
LAD: left anterior descending coronary artery
LV: left ventricle
LVESVI: left ventricle end-systolic volume index
MBF: myocardial blood flow
MCB: master cell bank
NOG, NOD/Shi-scid: IL-2R γnull
NYHA: New York Heart Association classification
PCI: percutaneous coronary intervention
peak VO_2_: peak oxygen uptake
PET: positron emission tomography
RCA: right coronary artery.
