# Supplementary material for: Case report: Transplantation of human induced pluripotent stem cell-derived cardiomyocyte patches for ischemic cardiomyopathy
Source: Front Cardiovasc Med. 2022 Aug 16;9:950829. doi: 10.3389/fcvm.2022.950829 (PMC9426776; doi:10.3389/fcvm.2022.950829)
Supplement: Supplementary figure 1 — Characteristic properties of hiPSC-CM patch. [file Data_Sheet_1.pdf]

Supplementary Material

1 Supplementary Methods

1.1 hiPSC Culture and Cardiomyogenic Differentiation and Purification

The methods for establishing clinical-grade hiPSCs and generation of master cell bank (MCB) of the hiPSC-cell line (QHJI14s04) have been described elsewhere (1). Briefly, the clinical-grade iPS cell line (QHJI14s04) was established from the peripheral blood mononuclear cells of a healthy donor (HLA homozygous: HLA-A, HLA-B, HLA-DRB1), showing the most frequent haplotypes in the Japanese population (2). QHJI14s04 was generated using episomal plasmids (pCE-hSK, pCE-hUL, pCE-hOCT3/4, pCE-mp53DD, pCXB-EBNA1) and maintained using a feeder-free and xeno-free culture system (3) in the cell processing center (Facility for iPS Cell Therapy, CiRA). The evaluation method for ensuring quality and the results are reported separately (1). For the production of hiPSC-CMs, the MCB of QHJI14s04 was established under good manufacturing practice conditions.

The QHJI14s04 cells from MCB were cultured on iMatrix511 (Nippi, Tokyo, Japan) coated dishes in Stem Fit Ak03N (Ajinomoto, Tokyo, Japan). Cardiomyogenic differentiation of QHJI14s04 cells was induced using a previously described protocol (3-5).

The methods used for evaluating the safety of the manufactured hiPSC-CMs, including assays for general toxicity and determination of *in vitro* and *in vivo* tumorigenicity via cell growth assay, soft agar colony formation assay, whole-genome/whole-exome sequencing analysis, and transplantation of the hiPSC-CMs into severe immunodeficient (NOG) mice, have also been described separately (1).

1.2 Cell Patch Preparation

The hiPSC-CM patches were manufactured using temperature-responsive culture dishes (6). Prior to cell seeding, the surface of the temperature-responsive dishes (UpCell; CellSeed, Japan) was coated with FBS overnight. After freeze–thawing, cells were plated onto the UpCell in DMEM containing 20% FBS and cultured at 37 °C under 5% CO_2. After 48 h in culture, hiPSC-CM patches were harvested and washed gently with Hanks' Balanced Salt Solution (+).

1.3 Flow Cytometry

hiPSC-CMs were labeled with anti-cardiac troponin T (cTnT) (REA400, Miltenyi Biotec, Germany) antibodies after fixation with paraformaldehyde. Cell populations (more than 10000 cells) were resolved using the MACSQuant Analyzer (Miltenyi Biotec). Data were analyzed using the FlowJo (BD Biosciences) software.

1.4 Hematoxylin and Eosin (H&E) and Immunofluorescence Staining

hiPSC-CM patches were fixed in 10% buffered formalin (Fujifilm), paraffin-embedded, and sectioned. The sections were stained with H&E (Muto Pure Chemicals). Immunofluorescence staining was performed with primary antibodies [anti-cTnT (MA5-12960, Neomarkers, Inc, Portsmouth, NH, USA) and anti-connexin 43 (C6219, Sigma-Aldrich, St. Louis, MO, USA)] overnight at 4 °C. Then the sections were labeled with secondary antibodies [Alexa Fluor 488 goat anti-rabbit A11008, and Alexa Flour 555 goat anti-mouse A21422 (both from Thermo Fisher Scientific, Waltham, MA, USA)] at room temperature for an hour. The cell nuclei were stained with Hoechst 33342 (1:100; Dojindo, Kumamoto, Japan), and the preparations were assessed using the confocal laser scanning microscope FV10i (Olympus, Tokyo, Japan). The images were analyzed using the FV10-ASW 3.1 software (Olympus).

1.5 *In vivo* Tumorigenicity Assay

The production lot of the hiPSC-CM used in the clinical trial was subjected to an *in vivo* tumorigenicity test and confirmed to be non-tumorigenic in immunodeficient NOG mice. The detailed procedures of transplantation and histological analysis have been described separately (1). Mice were euthanized and dissected 16 weeks after transplantation, and the major organs and tissues were carefully observed; gross pathological findings were recorded, and the tissues were stored for histological analysis.

1.6 Properties of hiPSC-CM Patches

The detailed characterization of the hiPSCs and the MCB are presented elsewhere (1) and in the Supplementary Data (Supplementary Table 1), respectively. The hiPSC-CM patch used in this clinical trial was prepared by a method that cleared the tumorigenicity denial test earlier (1). The hiPSC-CM cells used passed all quality inspections, as shown in Supplementary Table 1.

After preparing the myocardial patch, it was washed to remove as much medium components such as FBS as possible.

Safety of the patches was assessed *in vivo* using NOG mice. A patch prepared from cells of the same lot as the hiPSC-CMs used in the clinical trial was transplanted into NOG mice and observed for 16 weeks; neither teratomas nor malignant tumors were observed (Supplementary Table 1). Based on these data, we concluded that the hiPSC-CM patch used in the clinical trial had no risk of tumorigenesis following transplantation into the patient.

hiPSC-CMs were positive for the marker cTnT (Supplementary Figure 1A, Supplementary Table 1). Prior to transplantation surgery, hiPSC-CM patches were prepared using temperature-responsive culture dishes (Supplementary Figure 1B). The immunohistochemistry analysis of the patches revealed well-organized sarcomere structures and a high expression of the gap junction-related protein, connexin 43 (Supplementary Figure 1C).

To confirm the absence of neoplastic potential due to critical genomic mutations and survival of foreign genes, whole-genome sequencing analysis was performed on the MCB used in this study. No abnormalities in copy number variation (CNV) and single nucleotide polymorphism (SNP) were observed for cancer-related genes listed in the Catalogue of Somatic Mutations in Cancer (COSMIC), the Cancer Gene Census (1).

To verify whether genomic structural abnormalities are induced during the production process of hiPSC-CM patches, changes in CNV before and after production were evaluated by array comparative genomic hybridization (CGH) analysis using patch prepared from cells of the same lot as the hiPSC-CMs used in the clinical trial. No CNV abnormality was detected, and it was judged that no significant genomic structural mutation occurred during the manufacturing of the hiPSC-CM patches. CGH analysis showed that the cells produced for this clinical trial were also free of CNV abnormalities.

In order to detect teratoma-forming cells with the hiPSC-MC patch, mRNA expression of Lin28A, a marker of undifferentiated cells, was examined *in vitro* with hiPSC-CMs. As a result, Lin28A expression was below the detection limit (data not shown).

1.7 Assessment of Regional Myocardial Displacement Using Four-Dimensional Computed Tomography (4DCT)

The dynamic data from 320-slice cardiac CT images were restored at a constant interval from 10 phases via electrocardiogram synchronization. Subsequently, data complementation (30 phases) using PhyZiodynamics (PhyZiodynamics, 4D motion analysis; Ziosoft Inc., Tokyo, Japan) was performed as reported previously (7, 8). Next, a 4D motion analysis was performed to evaluate regional myocardial displacement. In the color scale, red indicates a good dynamic region, and the darker the color, the lower the movement.

1.8 End-Systolic Wall Stress (ESS)

Local ESS was calculated as previously reported (9) and using the Janz equation (10):

$$ESS = P \times \Delta Ac/\Delta Aw$$

where P is the LV end-systolic pressure, and ΔAc and ΔAw are the local cross-sectional areas of the LV cavity and the LV wall at end-systole in each log-axis plane, respectively. The cross-sectional wall area was the area bounded by two lines that are perpendicular to the cavity surface.

In this study, the LV end-systolic pressure estimate was obtained using the following equation (11):

$$P = 0.98 \times (\text{systolic blood pressure} + (2 \times \text{diastolic blood pressure}))/3 + 11 \text{ mm Hg}$$

Of note, the software can measure local ESS along 30 chords evenly spaced along the epicardial border in each image.

1.9 PET/CT

PET/CT images were acquired using the Biograph Vision 600 (SIEMENS Healthineers, Erlangen, Germany) in the 3-D mode (pixel size: 1.65 mm, slice thickness: 3 mm). FDG-PET scan was performed 60 min after injection of FDG (191 MBq) with glucose loading for heart and whole-body screening. For the evaluation of myocardial blood flow (MBF), a ^{13}N-ammonia PET scan was performed immediately after the injection of $^{13}N\text{-}NH_3$ (approximately 360 MBq) at rest and under stress (120 μg/kg/min of adenosine infusion over 6 min). PET images were reconstructed using the three-dimensional ordered subset expectation maximization algorithm with three iterations/five subsets and Gauss-filtered to a transaxial resolution of 3 mm at full-width at half-maximum.

Attenuation correction was performed using the unenhanced CT (120 kVp and 200 mAs). MBF was calculated using Cedars (Syngo software; version 5.1; Siemens Healthineers). Myocardial flow reserve was defined as the ratio of MBF at rest to MBF under stress.

2. Supplementary Figures

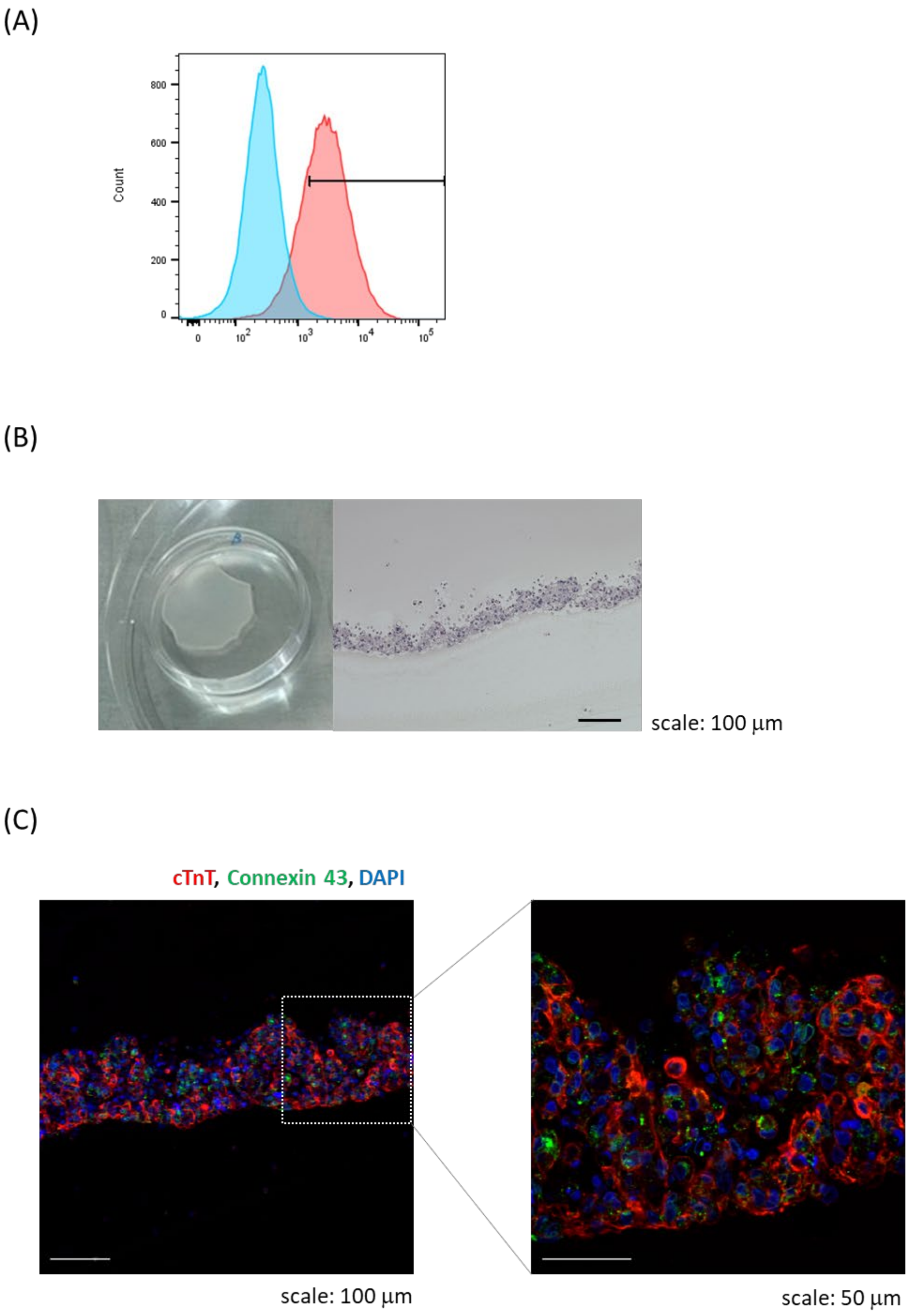

Supplementary Figure 1. Characteristic properties of hiPSC-CM patch.

A: Representative flow cytometry results for troponin T-positive cells (73.4%) in the hiPSC-CM patch. B: Appearance of hiPSC-CM patch and results of hematoxylin and eosin (H&E) staining. Left: Appearance of an iPSC-CM patch in 60 mm dish; Right: H&E staining of a hiPSC-CM patch (cross-section). Scale bar: 100 µm. C: Immunohistochemical staining of the hiPSC-CM patch (cross-section). Red: cTnT, green: connexin 43, blue: DAPI. Left: Low magnification image, scale bar: 100 µm; Right: High-magnification image, scale bar: 50 µm.

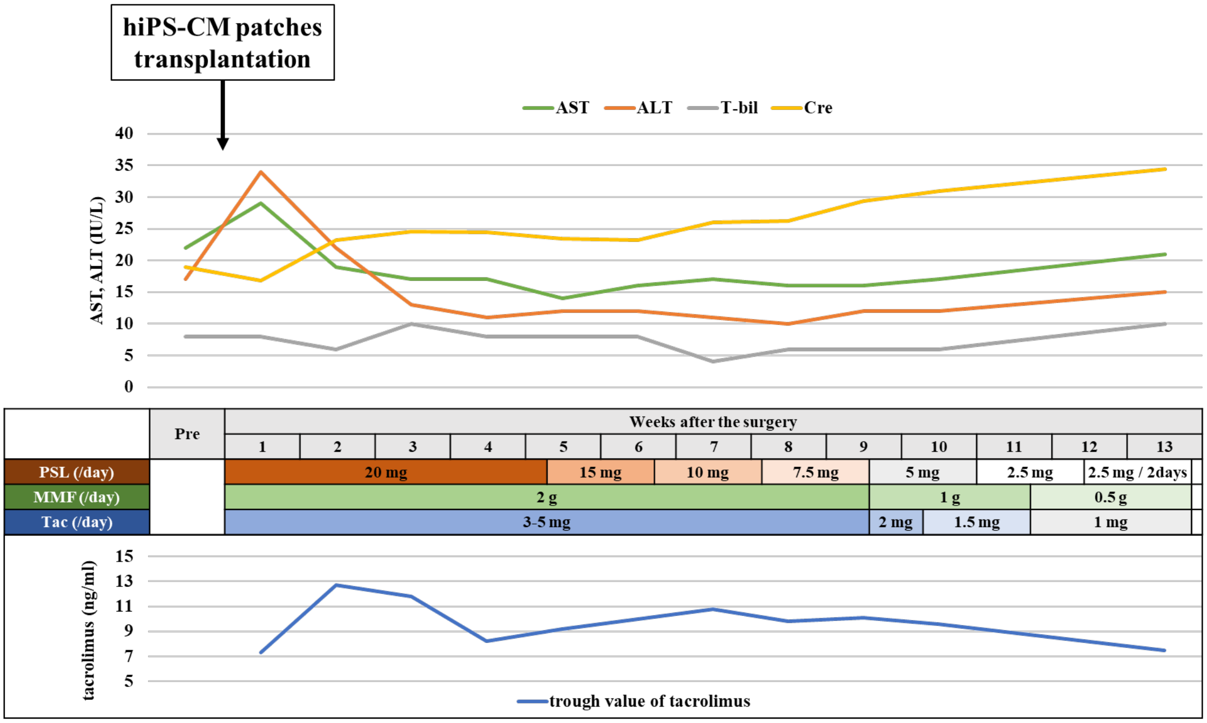

Supplementary Figure 2. Dose of immunosuppressants, the trough value of tacrolimus, and biomarker levels of renal and liver function.

The dose of immunosuppressants (PSL, MMF, and Tac;), the trough value of Tac, and the serial data of AST, ALT, T-bil, and Cre levels are demonstrated. The initial dose of PSL and MMF was 20 mg and 2 g per day, respectively, and the dose was gradually decreased over three months. The initial dose of Tac was determined by targeting its trough values in the range of 10 to 15 ng/ml, and it was also gradually reduced over three months. AST and ALT levels were elevated immediately after the surgery but soon returned to the normal range. T-bil level was maintained within the normal range during the follow-up period. However, Cre level gradually increased to 1.72 mg/dl, but the level dropped shortly after immunosuppressant suspension. PSL: prednisolone; MMF: mycophenolate mofetil; Tac: tacrolimus; AST: aspartate aminotransferase; ALT: alanine aminotransferase; T-bil: total bilirubin; Cre: creatinine; hiPSC-CM: human induced pluripotent stem cell-derived cardiomyocyte.

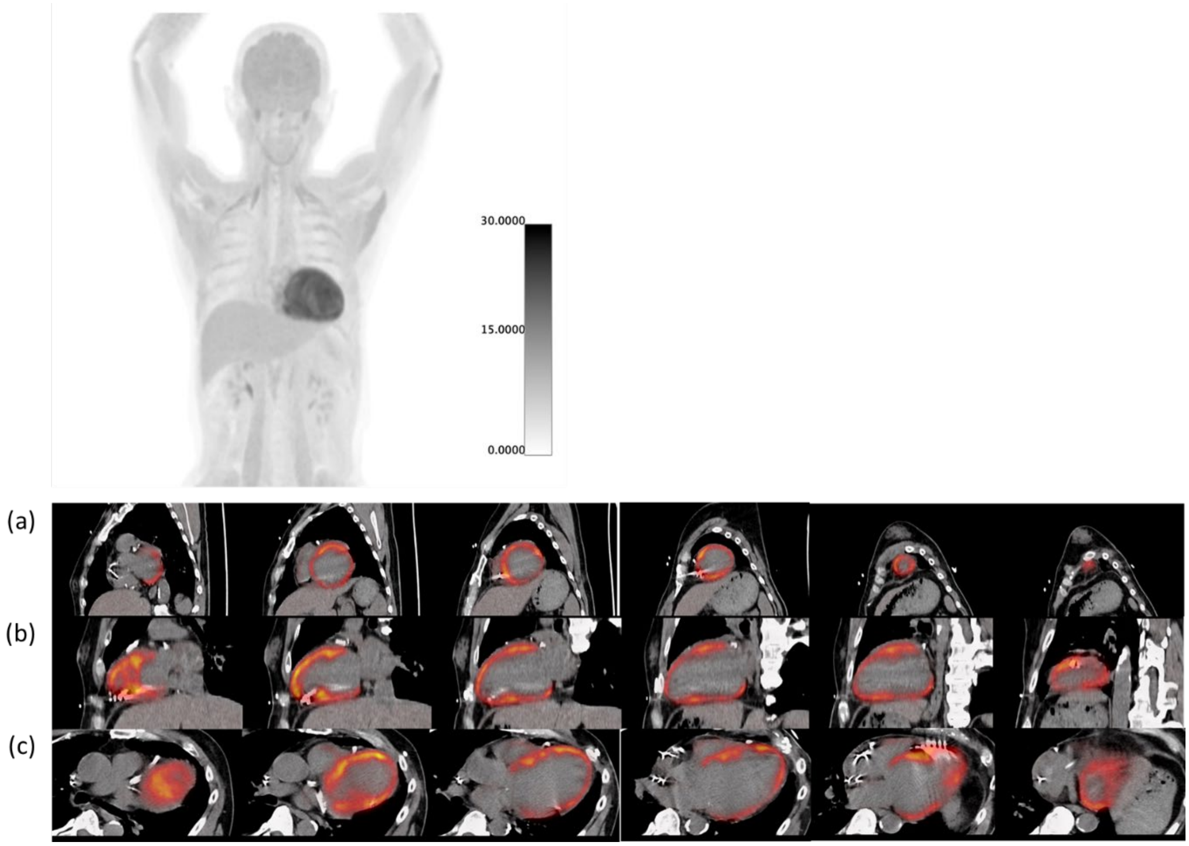

Supplementary Figure 3. FDG-PET imaging at 6 months after transplantation of hiPSC-CM patch.

FDG-PET showed no obvious abnormal accumulation in the whole-body (top panel: maximum intensity projection) and the whole heart (bottom panel: PET/computed tomography (CT) fusion of short axis (a), the long vertical axis (b), and long horizontal axis (c) views), indicating no tumorigenesis after the transplantation of human induced pluripotent stem cell-derived cardiomyocytes.

3. Supplementary Tables

Supplementary Table 1. Quality test of hiPSC-CMs.

Assay	Method	Results
Viability	Trypan Blue exclusion test	59.1%
Purity of cardiomyocytes	Flow cytometry	73.4%
Sterility testing	Membrane filtration method	Negative
Mycoplasma testing	Nested PCR	Negative
Endotoxin testing	Turbidimetric technique	< 0.194 EU/mL
in vivo tumorigenicity assay	Transplantation of hiPSC-CM patch onto the surface of the heart of NOG mice	No tumor formation in the mice tested (n=10)

The purity of cardiomyocytes was determined from the population of cTnT-positive cells measured by FACS. hiPSC-CM: human induced pluripotent stem cell-derived cardiomyocyte; NOG: NOD/Shi-scid, IL-2R γnull.

Supplementary Table 2. Changes in cardiac function, exercise tolerance, and heart failure classification after transplant surgery.

Time point	LVEF (%)	LVESVI (mL/m^2)	LVEDVI (mL/m^2)	peak VO_2 (mL/min/kg)	NYHA
pre	30.0	95.71	137.42	15.4	3
6 months	28.0	113.61	158.58	18.7	2
1 year	33.5	88.82	133.58	20.0	2

LVEF: left ventricular ejection fraction; LVESVI: left ventricular end-systolic volume index; LVEDVI: left ventricular end-diastolic volume index; peak VO_2: peak oxygen consumption; NHYA: New York Heart Association.

Supplementary references

1 Miyagawa S, Kawamura T, Ito E, Takeda M, Iseoka H, Yokoyama J, et al. Evaluation of the efficacy and safety of a clinical grade human induced pluripotent stem cell-derived cardiomyocyte patch: A pre-clinical study. bioRxiv [Preprint] (2021). Available at: https://doi.org/10.1101/2021.04.07.438744.

2 Okita K, Matsumura Y, Sato Y, Okada A, Morizane A, Okamoto S, et al. A more efficient method to generate integration-free human iPS cells. Nat Methods (2011) 8:409-12. doi: 10.1038/nmeth.1591.

3 Nakagawa M, Taniguchi Y, Senda S, Takizawa N, Ichisaka T, Asano K, et al. A novel efficient feeder-free culture system for the derivation of human induced pluripotent stem cells. Sci Rep (2014) 4:3594. doi: 10.1038/srep03594.

4 Ito E, Miyagawa S, Yoshida Y, Sawa Y. Efficient method to dissociate induced pluripotent stem cell-derived cardiomyocyte aggregates into single cells. Methods Mol Biol (2021) 2320:29-33. doi: 10.1007/978-1-0716-1484-6_4.

5 Ito E, Miyagawa S, Takeda M, Kawamura A, Harada A, Iseoka H, et al. Tumorigenicity assay essential for facilitating safety studies of hiPSC-derived cardiomyocytes for clinical application. Sci Rep (2019) 9:1881. doi: 10.1038/s41598-018-38325-5.

6 Kawamura M, Miyagawa S, Miki K, Saito A, Fukushima S, Higuchi T, et al. Feasibility, safety, and therapeutic efficacy of human induced pluripotent stem cell-derived cardiomyocyte sheets in a porcine ischemic cardiomyopathy model. Circulation (2012) 126(11) Suppl 1:S29-37. doi: 10.1161/CIRCULATIONAHA.111.084343.

7 Yoshida S, Miyagawa S, Toda K, Domae K, Sawa Y. Skeletal myoblast sheet transplantation enhanced regional improvement of cardiac function. Eur Heart J Cardiovasc Imaging (2018) 19:828-9. doi: 10.1093/ehjci/jey064.

8 Masumoto A, Kitai T, Ota M, Kim K, Ehara N, Furukawa Y. Real-time observation of a high-echoic mass in the left ventricle during transcatheter aortic valve implantation: A case report. Eur Heart J Case Rep (2020) 4:1-4. doi: 10.1093/ehjcr/ytaa392.

9 Takeda K, Taniguchi K, Shudo Y, Kainuma S, Hamada S, Matsue H, et al. Mechanism of beneficial effects of restrictive mitral annuloplasty in patients with dilated cardiomyopathy and functional mitral regurgitation. Circulation (2010) 122(11) Suppl:S3-9. doi: 10.1161/CIRCULATIONAHA.109.927855.

10 Janz RF. Estimation of local myocardial stress. Am J Physiol (1982) 242:H875-81. doi: 10.1152/ajpheart.1982.242.5.H875.

11 Rozich JD, Carabello BA, Usher BW, Kratz JM, Bell AE, Zile MR. Mitral valve replacement with and without chordal preservation in patients with chronic mitral regurgitation. Mechanisms for differences in postoperative ejection performance. Circulation (1992) 86:1718-26. doi: 10.1161/01.cir.86.6.1718.
